# *Drosophila* muscles regulate the immune response against wasp infection via carbohydrate metabolism

**DOI:** 10.1038/s41598-017-15940-2

**Published:** 2017-11-16

**Authors:** Hairu Yang, Dan Hultmark

**Affiliations:** 1 Department of Molecular Biology, Umeå University, S-901 87 Umeå, Sweden; 2 Institute of Biomedical Technology, University of Tampere, FI-33520 Tampere, Finland; 3 Present Address: Immunology Program, Memorial Sloan Kettering Cancer Center (MSKCC), New York, NY 10065 USA

## Abstract

We recently found that JAK/STAT signaling in skeletal muscles is important for the immune response of *Drosophila* larvae against wasp infection, but it was not clear how muscles could affect the immune response. Here we show that insulin signaling is required in muscles, but not in fat body or hemocytes, during larval development for an efficient encapsulation response and for the formation of lamellocytes. This effect requires TOR signaling. We show that muscle tissue affects the immune response by acting as a master regulator of carbohydrate metabolism in the infected animal, via JAK/STAT and insulin signaling in the muscles, and that there is indirect positive feedback between JAK/STAT and insulin signaling in the muscles. Specifically, stimulation of JAK/STAT signaling in the muscles can rescue the deficient immune response when insulin signaling is suppressed. Our results shed new light on the interaction between metabolism, immunity, and tissue communication.

## Introduction

The immune response in *Drosophila* is specifically adapted to different kinds of infecting organisms. Bacteria and fungi induce a humoral immune response, in which antimicrobial peptides are induced via Toll or Imd signaling^1–4^. Larger pathogens, such as eggs laid by the parasitoid wasp *Leptopilina boulardi*, activate a cellular immune response^5–7^ that involves three hemocyte classes: plasmatocytes, crystal cells, and lamellocytes. Around 6–10 h after infection, plasmatocytes begin to recognize and bind to the wasp egg^8,9^. Simultaneously, a new class of cells appears in circulation, the lamelloblasts, which later differentiate to become circulating lamellocytes. The lamellocytes participate in forming a capsule around the parasite, together with the plasmatocytes, which meanwhile also undergo transformation into a lamellocyte-like state (lamellocytes, type II)^9^. Finally, phenoloxidases from crystal cells and lamellocytes produce melanin, which is deposited in the capsule^10,11^.

We have recently shown that the encapsulation response is further modulated by interactions between hemocytes and other larval tissues, such as fat body and muscles^12–14^. The cytokines Unpaired 2 and 3 (Upd2 and Upd3), are upregulated in the hemocytes of wasp-infected larvae, and within eight hours after infection the skeletal muscles respond by activation of the JAK/STAT (Janus kinase/signal transducers and activators of transcription) signaling pathway. We found that a functional JAK/STAT signaling in the muscles was a prerequisite for an efficient immune response^12^. In *Drosophila*, JAK/STAT signaling involves three cytokines (Upd1–3), one cytokine receptor (Domeless), one tyrosine kinase (Hopscotch), and one transcription factor (Stat92E)^15–17^. Loss-of-function mutations in the corresponding genes reduce the encapsulation response against wasp infection^12,18^, while artificial activation of this signaling triggers a cellular immune response, including lamellocyte formation and generation of melanized nodules, in a way that is reminiscent of the encapsulation response^19–24^.

We have now further studied what happens in the muscles of wasp-infected larvae, and found that the infection has strong effects on insulin signaling and glycogen storage in this tissue. The insulin signaling pathway is conserved between invertebrates and vertebrates^25^. In *Drosophila*, the secretion of eight identified insulin-like peptides (Ilps) depends on the developmental stage, type of tissue, and environmental factors^26,27^. Secreted Ilps bind to the single insulin-like receptor (InR) in the target tissues, activating the downstream components sequentially, including a phophoinositide 3 kinase (Pi3K92E), an AKT homolog (Akt1), Target of rapamycin (Tor) and forkhead box, sub-group O (Foxo). Finally activated insulin signaling exerts its effect on different biological processes, for instance growth, development, metabolism, behavior, life span and immunity^28–30^. Besides the classical insulin pathway, *Drosophila* has two homologs to the relaxin receptor, Lgr3 and Lgr4, of which at least Lgr3 can serve as a receptor for the insulin-like peptide Ilp8^31–33^.

Our results suggest that JAK/STAT signaling in the muscles has profound effects on insulin signaling in the entire organism, that insulin in turn gives positive feedback on the JAK/STAT response, and that *Drosophila* larval skeletal muscles have a surprising role in the cellular immune response against wasp infection by controlling carbohydrate metabolism and feeding behavior.

## Results

### Insulin signaling in muscles is required for cellular immune response against wasp infection

**Figure 1:**
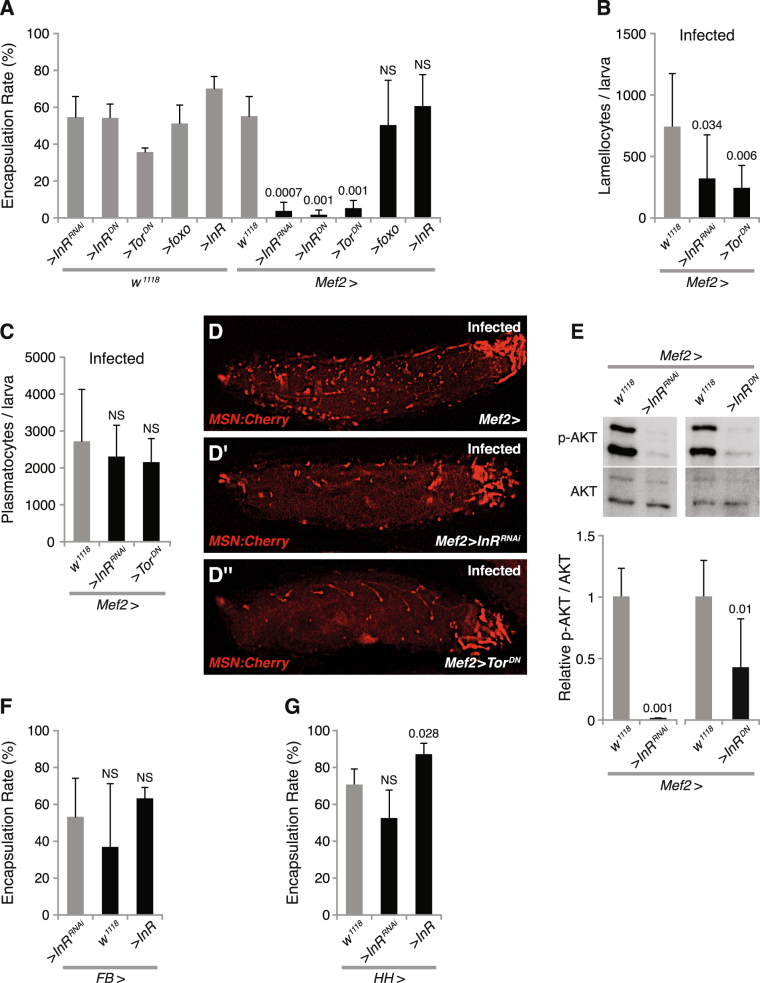
PI3K-AKT/TOR signaling in muscles is required for a cellular immune response against wasp infection. (**A**) Encapsulation rate when affecting insulin signaling, TOR signaling, and Foxo signaling in skeletal muscles with indicated genotypes. (**B**,**C**) Number of lamellocytes (**B**) and plasmatocytes (**C**) per larva after 12 h wasp infection, when suppressing PI3K signaling or TOR signaling with the indicated genetic constructs. (**D,D”**) *msn-Cherry* – labeled lamellocytes in larvae after 12 h wasp infection, when suppressing insulin or TOR signaling with the indicated genetic constructs. (**E**) Immunostaining of phospho-AKT from larval skeletal muscles, when suppressing insulin signaling by expressing *InR* ^*RNAi*^ or *InR* ^*DN*^ with *Mef2-GAL4*. Quantification of the immunostained bands is shown in the lower panel. (**F**) Encapsulation rates when suppressing or activating PI3K signaling in the fat body with the indicated genetic constructs. (**G**) Encapsulation rate when suppressing or activating PI3K signaling in hemocytes with the indicated genetic constructs. Data information: Encapsulation rates were determined in at least three independent experiments, and in total at least 100 larvae were analyzed. For hemocyte counts, at least eight larvae were analyzed for each genotype. Bars show averages and standard deviations. For immunostaining quantification, at least three independent experiments were analyzed. Bars show the average amounts of phospho-AKT, normalized to total AKT, and standard deviations. The *P*-values (unpaired *t*-test, unequal variance) are indicated, NS: not significant.

To investigate which activities, besides JAK/STAT signaling, are required in the muscles for the immune responses against wasp infection, we individually suppressed several well-known immune-related signaling pathways, including the p38, c-Jun N-terminal kinase (JNK), Toll, and insulin signaling pathways. We suppressed these pathways by expressing either RNA interference or dominant-negative constructs with a muscle-specific driver^34^, *Mef2-GAL4*, and then assayed encapsulation rates and hemocyte numbers. We found that suppression of insulin signaling in the muscles, by expressing *InR* ^*RNAi*^ or *InR* ^*DN*^, significantly reduced the encapsulation rate (Fig. 1A), while suppression of the other tested signaling pathways neither affected the encapsulation rate nor the number of lamellocytes and plasmatocytes in circulation (see Supplementary Fig. S1). Suppression of insulin signaling with *InR* ^*RNAi*^ also reduced the number of circulating lamellocytes (Fig. 1B), but not the number of circulating plasmatocytes (Fig. 1C). Taken together, these results indicate that insulin signaling is required in larval skeletal muscles for an efficient cellular immune response. To exclude the possibility that the decreased number of lamellocytes was due to attachment of these cells to other tissues, we used the *msn-Cherry* (*MSNF9mo-mCherry*) strain^35^ to monitor the lamellocytes *in vivo*. The *msn-Cherry* fluorescent reporter specifically labels lamellocytes, certain muscles and a few other tissues. Using this reporter, we confirmed that the number of lamellocytes was decreased in *InR*-suppressed larvae. The remaining *msn-Cherry* expression in these animals is essentially limited to background ectopic expression of this reporter in the feeding apparatus and certain tracheal branches (compare Fig. 1D and D’). Immunoblotting confirmed that expression of *InR* ^*RNAi*^ or *InR* ^*DN*^ in muscles significantly reduced phosphorylated AKT in muscles, indicating that these two constructs can successfully block insulin signaling in muscles (Fig. 1E).

Tor and Foxo are two major downstream targets of AKT signaling, Tor being a positive and Foxo a negative mediator of insulin signaling^25,36^. To investigate which of these two branches of the insulin signaling pathway mediate the effects of AKT in the muscles, we tested the effect on encapsulation rate when we suppressed *Tor* or overexpressed *foxo* in muscles. Our results showed that suppressing *Tor* in skeletal muscles, by expressing *Tor* ^*DN*^, significantly reduced the encapsulation rate and lamellocyte numbers (Fig. 1A,B and D”), but we saw no effect on the number of plasmatocytes in circulation (Fig. 1C). By contrast, we did not observe any obvious effect on the encapsulation rate when we overexpressed *foxo* (Fig. 1A). Taken together, these results suggest that, besides JAK/STAT signaling, insulin signaling is also necessary in muscles for the encapsulation response, and that this is mediated by signaling via Tor. These results further underscore the importance of *Drosophila* skeletal muscles in cellular immune response during wasp infection.

Considering that insulin signaling in the fat body plays an important role for the control of general metabolism, and that the fat body is also an immune responsive tissue^2,37,38^, we next tested the role of insulin signaling in the fat body on the encapsulation response. However, silencing or overexpressing *InR* in the fat body with a fat body-specific driver, *FB-GAL4* ^39^, did not significantly affect the encapsulation rate (Fig. 1F), indicating that insulin signaling in the fat body is not essential for the immune response against wasp infection. Similarly, since hemocytes are main players in the encapsulation response, we also investigated whether insulin signaling in hemocytes affect this response. For this purpose, we expressed *InR* ^*RNAi*^ in hemocytes with a combination (“*HH-GAL4*”)^13^ of two hemocyte drivers, *He-GAL4* and *Hml* ^*Δ*^ *-GAL4*, but again we could not observe any effect on the encapsulation rate (Fig. 1G). However, overexpression of *InR* in hemocytes slightly improved the encapsulation rate, from 71% to 81% (Fig. 1G), but altogether these results suggest that insulin signaling is neither essential in fat body nor in hemocytes for the encapsulation response. We also tested the effect of suppressing p38, JNK and Toll signaling in fat body, but neither of these treatments significantly affected the encapsulation rate, or the number of hemocytes (see Supplementary Fig. S2).

### Wasp infection affects both insulin and JAK/STAT signaling

**Figure 2:**
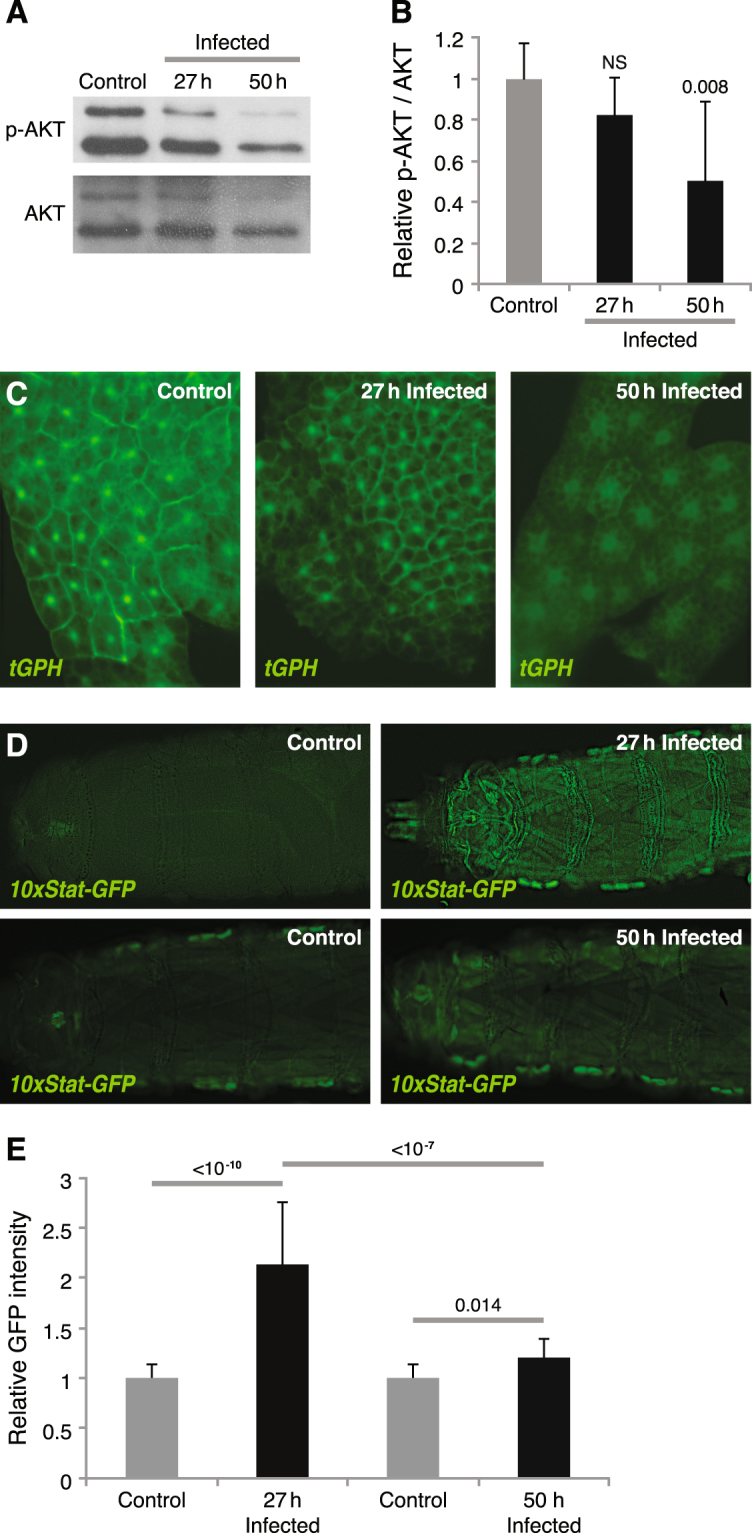
Wasp infection affects insulin and JAK/STAT signaling. (**A**) Western blot, showing the amount of phospho-AKT in larval skeletal muscles after 27 or 50 h wasp infection. (**B**) Quantification of immunostained bands after 27 or 50 h wasp infection. (**C**) Insulin signaling activity in larval fat body detected by *tGPH* reporter after 27 or 50 h wasp infection. (**D**) JAK/STAT signaling activity in larval skeletal muscles detected by the *10XStat-GFP* reporter after 27 or 50 h wasp infection. (**E**) Quantification of GFP signaling in muscles. Data information: For immunostaining quantification, at least three independent experiments were analyzed. Bars show the average amounts of phospho-AKT normalized to total AKT, and standard deviations. For GFP quantification, at least 10 larvae were quantified. Bars show averages and standard deviations. The *P*-values (unpaired *t*-test, unequal variance) are indicated, NS: not significant.

Since we found that an intact insulin signaling pathway was required in the skeletal muscles for a successful encapsulation response, we next investigated how wasp infection affects insulin signaling activity in muscles and elsewhere. Using immunoblotting, we found that phosphorylated AKT was significantly reduced in muscles 50 hours after wasp infection, but we saw no significant difference at the 27-hour time point (Fig. 2A and B), indicating that a longer period of wasp infection leads to reduced insulin signaling activity in skeletal muscles. Furthermore, we tested whether wasp infection also affects insulin signaling activity in the fat body. For this purpose, we used the *tGPH* construct as a reporter for insulin signaling. This construct encodes green fluorescent protein (GFP) fused to a pleckstrin homology (PH) domain. In response to insulin signaling the GFP-PH fusion protein becomes redirected to the cell membrane, due to the affinity of the PH domain for phosphatidylinositol (3,4,5)-trisphosphate. The localization of this reporter is not distinct in muscles, but it works well in the fat body^40^. We found that GFP was localized at the cell membrane, both in uninfected larvae and 27 hours after wasp infection. However, 50 hours after wasp infection GFP became enriched in the cytoplasm, suggesting that, just like in the muscles, insulin activity is reduced in the fat body after a longer period of wasp infection (Fig. 2C).

Previously we found that JAK/STAT signaling activity is induced in skeletal muscles as early as 8 hours after wasp infection and is maintained at a high activity until 27 hours after wasp infection^12^. We have now further monitored JAK/STAT activity with the *10xStat-GFP* reporter up to 50 hours after wasp infection. As shown in Fig. 2D and E, we found that GFP expression in skeletal muscles was reduced by 50 hours after wasp infection when compared with the 27-hour time point, though it is still higher than in the uninfected control, indicating that a longer period of wasp infection reduces the wasp infection-induced activation of JAK/STAT signaling in skeletal muscles.

### JAK/STAT signaling in muscles regulates insulin signaling systemically

**Figure 3:**
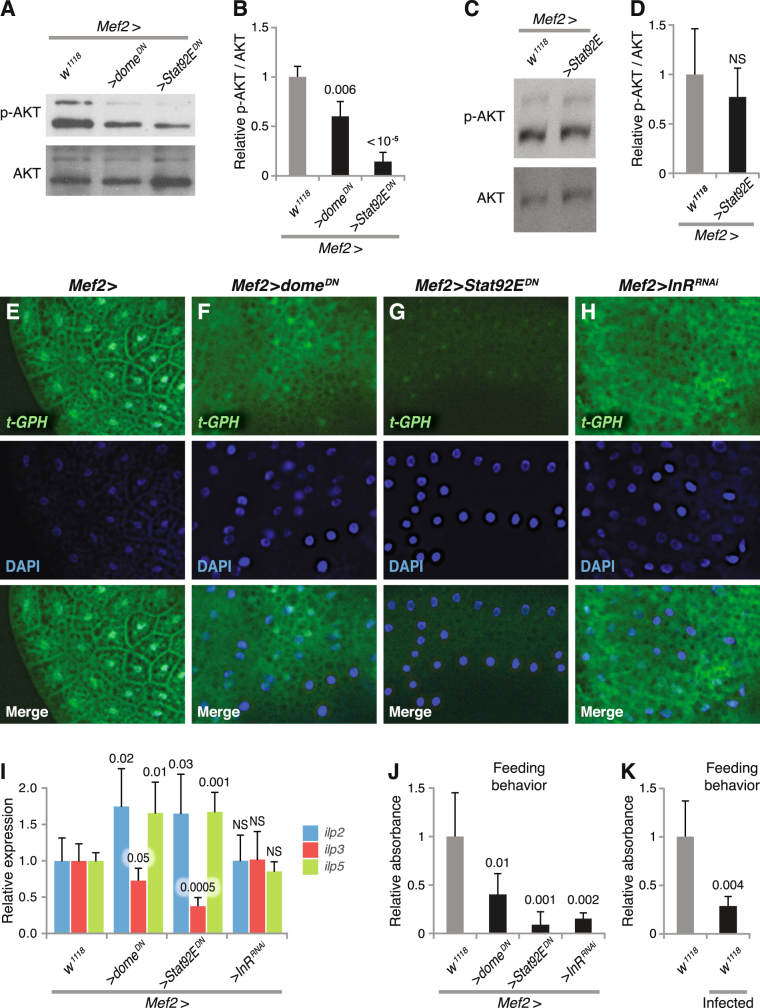
JAK/STAT signaling regulates insulin signaling systemically. (**A**,**B**) Amount of phospho-AKT in larval skeletal muscles when suppressing JAK/STAT signaling by expressing *Stat92E* ^*DN*^ or *dome* ^*DN*^, assayed by western blotting. (**C**,**D**) Amount of phospho-AKT in larval skeletal muscles when activating JAK/STAT signaling by overexpressing wild-type *Stat92E*, assayed by western blotting. (**E**–**H**) Insulin signaling activity in larval fat body visualized by *tGPH* reporter in control (**E**) or after suppressing JAK/STAT (**F**,**G**) or insulin signaling (**H**) in muscles with the indicated genetic constructs. Localization of GFP to the cell membrane indicates a high level of insulin signaling activity. (**I**) *ilp2, ilp3*, and *ilp5* transcripts in larval brain when suppressing JAK/STAT or insulin signaling in muscles. (**J**,**K**) Feeding activity when suppressing insulin and JAK/STAT signaling (**J**), or after 50 h wasp infection (**K**). Data information: For immunostaining quantification, at least three independent experiments were analyzed. Bars show the average amounts of phospho-AKT, normalized to total AKT, and standard deviations. For feeding behavior, at least three independent experiments were performed. Bars show averages and standard deviations. The *P*-values (unpaired *t*-test, unequal variance) are indicated, NS: not significant.

Suppression of insulin (Fig. 1A–D) or JAK/STAT signaling^12^ in *Drosophila* larval skeletal muscles generated similar phenotypes, with reduced lamellocyte production and a compromised encapsulation response against wasp infection. Therefore, we suspected that these two signaling pathways might interact with each other. First, we investigated whether JAK/STAT signaling affects insulin signaling in the skeletal muscles of uninfected larvae. For this purpose, we suppressed JAK/STAT signaling in muscles by expressing the dominant-negative *Stat92E* ^*DN*^ or *dome* ^*DN*^ constructs with the *Mef2-GAL4* driver. As shown in Fig. 3A and B, this reduced the level of phosphorylated AKT in the muscles, suggesting a reduced level of insulin signaling activity. On the other hand, activation of JAK/STAT signaling by overexpression of *Stat92E* with the *Mef2-GAL4* driver did not further increase AKT phosphorylation (Fig. 3C and D). Taken together, these results suggest that JAK/STAT signaling is required in skeletal muscles for a normal level of insulin activity.

This raised the question whether JAK/STAT signaling affects the insulin response only locally, in the skeletal muscles, or if insulin signaling is systemically affected also in other insulin-sensitive tissues, for example the fat body. To test this, we suppressed JAK/STAT signaling in the skeletal muscles by the same strategies as above, and monitored insulin activity in the fat body with the *tGPH* reporter. We found that expression of *Stat92E* ^*DN*^ or *dome* ^*DN*^ in muscles abolished GFP localization to the cell membrane in the fat body (Fig. 3E,F, and G), indicating that insulin signaling is also reduced in the fat body when JAK/STAT signaling is suppressed in skeletal muscles. Besides, insulin signaling was also reduced in the fat body when we suppressed insulin signaling in muscles (Fig. 3H).

Three insulin-like peptides, Ilp2, Ilp3 and Ilp5, are produced in a cluster of neurosecretory cells in the brain, and they are believed to be particularly important in the regulation of metabolism in *Drosophila* ^26^. We found that *ilp3* expression was substantially reduced in the brain when we suppressed JAK/STAT signaling in the muscles, while *ilp2* and *ilp5* expression was increased (Fig. 3I). This observation is in agreement with the recent finding that *ilp3* is specifically dedicated to the systemic control of circulating sugars, while *ilp2* responds to amino acids^41^. Surprisingly, although the phenotypic effects of suppressed insulin signaling in the muscles otherwise closely mimic those of JAK/STAT suppression, *InR* suppression in muscles had no significant effect on the expression of any of the three insulin-like peptides in the brain (Fig. 3I). It is possible that the systemic effects of this genotype are mediated by other insulin-like peptides elsewhere. To control the specificity of our qPCR assays, we repeated them with a second set of primer pairs for each of the three *ilp* genes, with virtually identical results. Figure 3I is based on the mean values of the two assays for the individual samples. Taken together, these results suggest that suppression of JAK/STAT signaling in the muscles has a systemic effect on insulin signaling, probably in the entire organism, and that interference with insulin signaling in the muscles has a similar feedback effect.

Next, we investigated the mechanism by which JAK/STAT signaling may affect insulin activity. It has previously been reported that insulin signaling in *Drosophila* larval muscles affects body size, probably by regulating feeding behavior^42^. Thus, we suspected that the systemically reduced insulin activity after suppressing JAK/STAT signaling in larval skeletal muscles was due to decreased feeding activity. To test this idea, we assayed the larval food intake by measuring the intake of food dye, Brilliant Blue FCF. In agreement with the published reports, we found that suppression of insulin signaling in larval muscles reduced the intake of blue dye, indicating reduced feeding activity (Fig. 3J). More importantly, we observed a reduced feeding behavior also when we suppressed JAK/STAT signaling in skeletal muscles (Fig. 3J). In *Drosophila*, it has been shown that decreased nutrient availability reduces insulin signaling^36^. Altogether, these results indicate that blocking JAK/STAT signaling in skeletal muscles may have affected insulin signaling systemically, by affecting feeding activity and *ilp3* expression.

Since wasp infection reduces insulin signaling in both muscles and fat body, we hypothesized that wasp infection might also reduce feeding activity. We measured the food intake 50 hours after wasp infection, and as expected it was significantly reduced (Fig. 3K). This could explain the reduced insulin activity during wasp infection.

### Insulin signaling in *Drosophila* skeletal muscles regulates JAK/STAT signaling

**Figure 4:**
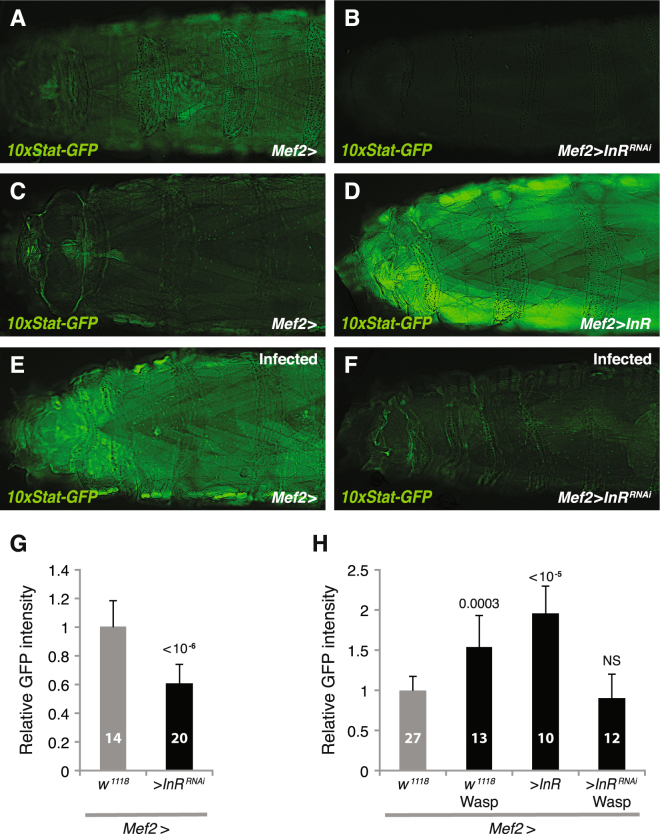
Insulin signaling in muscles regulates JAK/STAT signaling. (**A**–**F**) JAK/STAT signaling activity in larval skeletal muscles, detected by the *10xStat-GFP* reporter, when suppressing or activating insulin signaling by expressing *InR* ^*RNAi*^ or wild type *InR*, respectively (**A**–**D**), or after 27 h wasp infection, when suppressing insulin signaling by expressing *InR* ^*RNAi*^ (**E**,**F**). (**G**,**H**) Quantification of GFP signaling in muscles. Data information: For GFP quantification, at least 10 larvae were quantified. Bars show averages and standard deviations. The *P*-values (unpaired *t*-test, unequal variance) are indicated, NS: not significant.

Next, we investigated whether insulin signaling also regulates JAK/STAT signaling in muscles. We artificially activated or suppressed insulin signaling in muscles by expressing *InR* ^*RNAi*^ or wild type *InR*, and monitored the resulting JAK/STAT activity with the *10xStat-GFP* reporter. Interestingly, we found that knockdown of *InR* in muscles reduced GFP expression from this reporter (compare Fig. 4A and B; quantified in Fig. 4G), indicating a reduced baseline JAK/STAT activity when insulin signaling is suppressed in muscles. Conversely, overexpression of wild type *InR* in muscles led to increased JAK/STAT activity (compare Fig. 4C and D; quantified in Fig. 4H). These results suggest that insulin signaling positively regulates JAK/STAT signaling in muscles. In addition, in line with the above results, we found that expression of *InR* ^*RNAi*^ in muscles blocked the wasp-induced JAK/STAT activation (compare Fig. 4E and F; quantified in Fig. 4H). Altogether, we conclude that insulin signaling can positively regulate JAK/STAT signaling in *Drosophila* skeletal muscles.

### Reciprocal interaction between JAK/STAT and insulin signaling in the immune response

**Figure 5:**
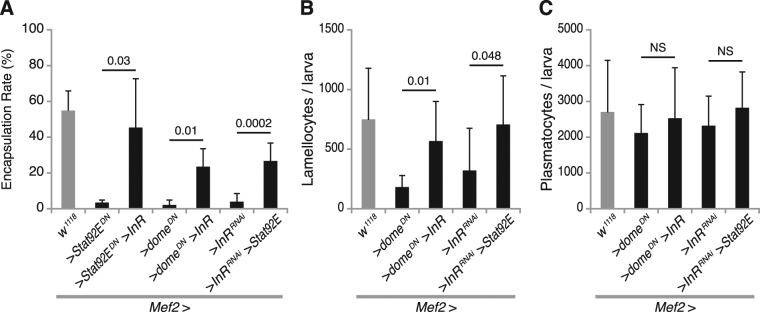
Mutual rescue by the JAK/STAT and insulin signaling pathways. (**A**–**C**) Encapsulation rates (**A**), number of lamellocytes (**B**) and number of plasmatocytes (**C**) per larva, when activating insulin signaling and simultaneously suppressing JAK/STAT signaling, or vice versa, with the indicated genetic constructs. Data information: Encapsulation rates were determined in at least three independent experiments, and in total at least 100 larvae were analyzed. For hemocyte counts, at least eight larvae were analyzed for each genotype. Bars show averages and standard deviations. The *P*-values (unpaired *t*-test, unequal variance) are indicated, NS: not significant.

Since we had found a reciprocal positive interaction between the JAK/STAT and insulin signaling pathways in *Drosophila* muscles, we tested whether we could rescue the immune response when we had suppressed one of these two signaling pathways by stimulating the other pathway. We found that overexpression of wild type *InR* in the background of *Stat92E* ^*DN*^ or *dome* ^*DN*^ expression in muscles significantly improved the encapsulation response and lamellocyte formation, but had no obvious effect on the number of circulating plasmatocytes (Fig. 5A–C). Importantly, while stimulating JAK/STAT signaling in muscles does not normally enhance the immune response^12^, overexpression of wild type *Stat92E* can largely rescue the encapsulation response and lamellocyte numbers when *InR* is suppressed, again without obvious effect on the number of plasmatocytes in circulation (Fig. 5A–C). Thus, for the immune response, loss of one of these two signaling pathways in the muscles can be compensated by increased activity in the other pathway, further underscoring the fact that the JAK/STAT and insulin signaling pathways interact and mutually affect each other.

### Glycogen storage in skeletal muscles is required for cellular immune response

**Figure 6:**
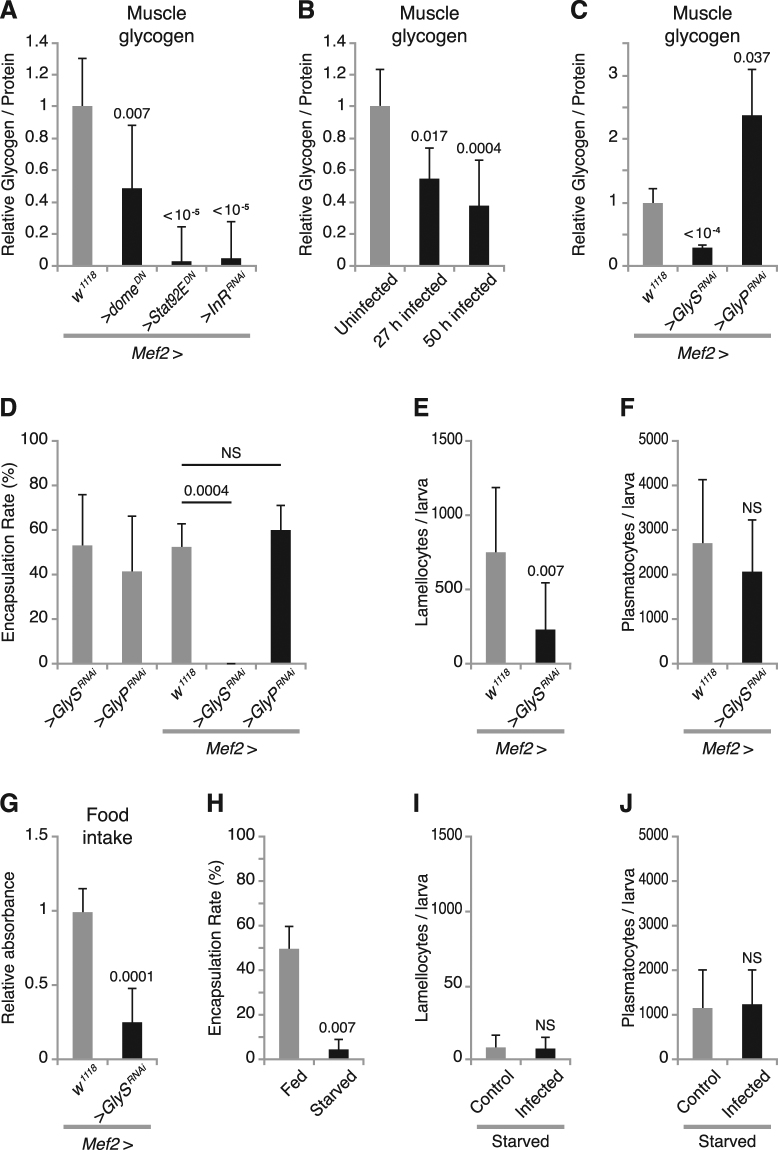
Glycogen storage is required for the cellular immune response against wasp infection. (**A**) Glycogen contents in skeletal muscles, normalized to protein, when suppressing JAK/STAT signaling and insulin signaling respectively in muscles with the indicated genetic constructs. (**B**) Glycogen contents in skeletal muscles normalized to protein, at different times after wasp infection. (**C**) Glycogen contents in skeletal muscles normalized to protein when suppressing glycogen synthase or glycogen phosphorylase in muscles by *UAS-GlyS* ^*RNAi*^ or *UAS-GlyP* ^*RNAi*^, respectively, with the *Mef2-GAL4* driver. (**D**) Encapsulation rates when suppressing glycogen synthase or glycogen phosphorylase in muscles by *UAS-GlyS* ^*RNAi*^ or *UAS-GlyP* ^*RNAi*^, respectively, with the *Mef2-GAL4* driver. (**E**,**F**) Number of lamellocytes (**E**) and plasmatocytes (**F**) per larva after 12 h wasp infection when suppressing glycogen synthase in muscles by *UAS-GlyS* ^*RNAi*^, with the *Mef2-GAL4* driver. (**G**) Feeding activity when depleting glycogen in muscles by expressing *UAS-GlyS* ^*RNAi*^ with the *Mef2-GAL4* driver. (**H**) Encapsulation rates under starvation conditions. (**I**,**J**) Number of lamellocytes (**I**) and plasmatocytes (**J**) per larva after 12 h wasp infection under starvation conditions. Data information: Encapsulation rates were determined in at least three independent experiments, and in total at least 100 larvae were analyzed. For hemocyte counts, at least eight larvae were analyzed for each genotype. For glycogen measurements, at least three independent experiments were done. Bars show averages and standard deviations. The P-values (unpaired *t*-test, unequal variance) are indicated, NS: not significant.

The strong interaction between JAK/STAT and insulin responses in muscles, and the systemic effects of these responses on insulin signaling in other tissues, raised the question if general metabolism were also affected under these conditions. We first investigated lipid metabolism in whole animals when we suppressed JAK/STAT or insulin signaling in skeletal muscles with *UAS-dome* ^*DN*^, *UAS-Stat92E* ^*DN*^, *UAS-InR* ^*DN*^, or *UAS-InR* ^*RNAi*^. However, neither free triglyceride nor total triglyceride was obviously affected (see Supplementary Fig. S3). Next, we investigated if carbohydrate levels were affected, either in hemolymph or in skeletal muscles. We saw no effect on free glucose, but we found that trehalose concentration in hemolymph was significantly reduced when we suppressed JAK/STAT or insulin signaling in the muscles (see Supplementary Fig. S4). This indicates that JAK/STAT and insulin signaling in muscles affect carbohydrate metabolism in the entire organism. In *Drosophila* larvae the carbohydrate storage form, glycogen, is primarily found in the skeletal musculature^43,44^. We therefore also assayed the effect of these two signaling pathways on the glycogen stores in the muscles. Interestingly, we found that glycogen levels were drastically reduced in the muscles when we suppressed insulin or JAK/STAT signaling (Fig. 6A). Thus, JAK/STAT and insulin signaling in muscles both affect general carbohydrate metabolism. In agreement with the findings of Bajgar *et al*.^45^, the glycogen stores were also affected in wasp-infected larvae (Fig. 6B), but we saw no effect on free glucose or trehalose (see Supplementary Fig. S4).

We next investigated to what extent glycogen storage in muscles affects the cellular immune response against wasp infection. We artificially decreased or increased glycogen storage in muscles by suppressing glycogen synthase or glycogen phosphorylase by expressing *UAS-GlyS* ^*RNAi*^ or *UAS-GlyP* ^*RNAi*^, respectively with the *Mef2-GAL4* driver (Fig. 6C). We found that decreased glycogen storage in larval skeletal muscles dramatically reduced the encapsulation rate from around 50% in controls to 0% (Fig. 6D). However, there was no obvious effect on the encapsulation rate when glycogen storage was increased (Fig. 6D). Furthermore, we observed a decreased number of lamellocytes when the glycogen stores were depleted in the muscles, but there was no obvious effect on the number of plasmatocytes in circulation (Fig. 6E and F). Taken together, these results show that the glycogen reserves in the muscles are necessary for an efficient encapsulation response. To test whether the compromised immune response was due to a decreased food intake, which therefore led to malnutrition, we tested the larval feeding behavior when depleting the glycogen storage in muscles. We found that food intake was significantly reduced when the glycogen stores were depleted in the muscles, indicating that a sufficient supply of glycogen, as an energy source for the skeletal muscles, was required for normal feeding activity (Fig. 6G).

Since wasp infection generates a major shift in the carbohydrate metabolism, and since a reduced food intake also compromises the immune response against wasp infection, we speculated that starvation might on the other hand affect the immune response^45^. To test this, we infected starved larvae, and found that starvation significantly reduced the encapsulation rates compared with the normal diet control (Fig. 6H), suggesting that nutrition is an important factor for *Drosophila* encapsulation response. Lamellocytes are rare in healthy uninfected larvae. However, starvation alone could induce formation of a small number of lamellocytes, but wasp infection did not further increase the number of lamellocytes in the starved larvae (Fig. 6I), and the numbers remained much lower than in fed, infected larvae (Figs 1B and 6D). Furthermore, starvation alone reduced the number of circulating plasmatocytes, compared to normal diet controls (compare Fig. 6J to Figs 1C and 6F). These results further underscore that a well-fed condition is very important for an efficient cellular immune response against wasp infection.

## Discussion

We observed very similar physiological effects when we suppressed JAK/STAT or insulin signaling in the muscles of otherwise healthy *Drosophila* larvae. In both cases, we observed suppressed feeding behavior and carbohydrate accumulation in the muscles, and both manipulations interfered with the ability of the larvae to cope with a wasp infection. The effects were also connected in the sense that suppressed JAK/STAT signaling led to reduced insulin signaling, and vice versa. We conclude that the basal JAK/STAT activity in the muscles is important for the carbohydrate metabolism, not only in the muscles but in the entire organism. The role of the increased JAK/STAT signaling that was seen after wasp infection is still uncertain, but it should probably be interpreted in this context.

Glucose metabolism as a source of energy is crucial for the growth, survival, proliferation, and differentiation of activated immune cells in mammals^46^. In *Drosophila*, Bajgar *et al*. have shown that within 18 hours after wasp infection, up to one third of the recently ingested glucose was reallocated from other tissues to the hemocytes^45^. Considering that hemocytes represent a very small fraction of the larval biomass, their sugar consumption must be enormous. Under these conditions, the wasp-induced JAK/STAT response in the muscles may boost insulin signaling and help to redirect nutrients towards the needs of the immune cells. While muscle-specific stimulation of JAK/STAT signaling could not further enhance the immune response in well-fed larvae^12^, we have here shown that it rescues the immunodeficiency caused by suppressed insulin signaling.

The interactions between infection, insulin signaling, feeding behavior and immune responses involve energy and nutrients. There is evidence, from *Drosophila* and other systems, that nutrition, microbiome, metabolism, and immunity are all interconnected^45,47,48^. Previous studies have already shown that the energy supply is one of the most important factors affecting pathogen growth^47^. Indeed, the presence of a parasite creates a competition for available nutrients and a starvation-like state in the larva. Our findings suggest that a wasp-induced reduction of food intake is detrimental for the cellular immune responses to wasp infection. It seems that during wasp infection, the benefits of an adequate food intake outweigh those of a reduced food intake.

Several studies have shown that the control of immunity and metabolism are mutually interconnected, and that insulin signaling plays an important role in this interaction (reviewed in refs^28,29^). The interactions between pathogens and insulin signaling are complicated and, depending on the nature of the pathogen, the effects may go in opposite directions. Some studies have shown that infections reduce insulin signaling activity^49–51^. However, other studies show that some infections can activate insulin signaling, at least in *Drosophila* ^52^. Our results show that after a longer period of wasp infection, insulin signaling activity is reduced in both *Drosophila* larval skeletal muscles and fat body. These findings indicate that modulation of insulin signaling in response to infection is a general phenomenon in both vertebrates and invertebrates.

On the other hand, insulin signaling also regulates immune responses. Inactivation of PI3K/AKT signaling generally leads to reduced inflammatory and immune responses, as demonstrated in different animal models^53^. In *Drosophila*, numerous studies have demonstrated the importance of nutrient availability and insulin/TOR signaling for organismal growth in general and for hematopoiesis in particular, at least in the lymph gland^54–58^. Here, we have focused on the special role of muscles and the nutrient status in this tissue, in the context of wasp infection. We have shown that suppression of insulin signaling in larval skeletal muscles, but not in the fat body and hemocytes, blocks the encapsulation response almost entirely and reduces the number of lamellocytes, suggesting that insulin signaling is essential in the immune response to wasp infection. At the same time the number of circulating plasmatocytes is normal, showing that basic larval hematopoiesis is largely unaffected.

A mutually positive interaction between JAK/STAT and insulin signaling has also been observed in other systems. In mammals, there is evidence suggesting that JAK/STAT signaling can directly feed into the insulin signaling pathway. For example, when JAK2 is activated by hormones, such as growth hormone or leptin, it can mediate phosphorylation of the insulin receptor substrate and thereby activate insulin signaling^59,60^. Conversely, previous studies in mammals reported that insulin signaling positively regulates Stat3 and Stat5 in different contexts^61–63^. However, the literature regarding these two signaling interactions is complex and partly conflicting. For instance, it was also reported that insulin signaling negatively regulates Stat3 transcription in human melanoma cells^64^.

It is believed that infectious diseases are important driving forces for the natural selection of behaviors that improve fitness^65^. A loss of host appetite (anorexia) is a common and apparently evolutionarily conserved response to infection in both vertebrates and invertebrates^66–69^. In *Drosophila*, the effect of food intake on the host’s survival depends on the pathogen: decreased food intake increases host survival during *S. typhimurium*, *E. coli*, and *E. carotovora* infections, but compromises host survival during *L. monocytogenes* infection^65^. We found that dietary restriction significantly reduced the encapsulation response to wasp infection.

Our results demonstrate that in *Drosophila*, skeletal muscles play a central role in this interplay between nutrition and immunity. The level of insulin signaling in the muscles feeds back on the systemic control of metabolism, which in turn affects the ability of the immune defense to cope with a parasite infection. The unique role of muscles in this context was unexpected, but is probably due to the fact that muscles provide the main reservoir for stored glycogen which supplies energy required for larvae to hatch, feed, and crawl^43,44,70^. Muscle-specific expression of the insulin-processing enzyme neprilysin 4 was recently found to control insulin-like peptide abundance and food intake^71^, giving further support for the role of muscles in the systemic control of the nutrient balance. Similarly, in a recently published paper^72^, Zhao and Karpac show how signals from skeletal muscles control nutrient storage in adult *Drosophila*. However, unlike the situation in the infected larva, they found that it was primarily the storage of lipids in the fat body that was affected in the adult fly.

While our study was focused on the systemic interactions between the muscles and the immune cells, it is possible that muscles are also able to interact directly with hemocytes in the “pockets” where sessile plasmatocytes are situated in close contact with the underlying musculature^73,74^. In mammals, muscles and macrophages (which are similar to *Drosophila* plasmatocytes) cross-talk during muscle regeneration and aging^75^. Our results indicate that the immune system is closely interacting with *Drosophila* muscles, via JAK/STAT and insulin signaling. These interactions may also have wider implications for the phenomenon of muscle wasting in the context of local or systemic inflammation.

## Methods

### *Drosophila* genetics

*Drosophila melanogaster* was reared on mashed potato diet at room temperature, unless otherwise indicated. *Leptopilina boulardi* G486 were bred on *D. melanogaster* Canton S stock at room temperature, and adult wasps were maintained in apple juice agar vials at room temperature. The following *D. melanogaster* strains were used: *10XStat92E-GFP* (BL26197)^76^, *UAS-Stat92E* ^*DN*^ ^77^, *UAS-dome* ^*DN*^ (*UAS-dome* ^*ΔCYT*^)^78^, *MSN9mo-mCherry* (here called *msn-Cherry*)^35^, *UAS-InR* ^*RNAi*^ (VDRC-992), *UAS-InR* ^*DN*^ (BL-8252), *UAS-Tor* ^*DN*^ (BL-7013), *UAS-foxo* (BL-9575), *UAS-InR* (BL-8262), *UAS-Stat92E*, *UAS-GlyS* ^*RNAi*^ (BL-34930), *UAS-GlyP* ^*RNAi*^ (BL-33634). *UAS-Pvr* ^*DN*^ (BL-58431), *UAS-Jra* ^*DN*^ (BL-7217), *UAS-kay* ^*DN*^ (BL-7214), and *UAS-Toll* ^*RNAi*^ (VDRC-100078). The following Gal4 driver stocks were used: *Mef2-Gal4* ^79^, *Fb-Gal4* ^39^, *Hemese-Gal4* (*He-Gal4*, BL8699)^24^ and *Hml* ^*Δ*^ *-Gal4* (BL30139)^80^.

### Encapsulation rate assay

Larvae with black capsules was sorted out, and remaining larvae without obvious black capsules were dissected to check whether they were infected. Finally, we calculated the encapsulation rate by calculating the ratio of larvae with black capsule to the total number of infected larvae, as previously described^12^.

### Immobilization of larvae and imaging

Imaging was done as previously described^12^. Briefly, third instar larvae were immobilized by cold treatment in glycerol, imaged with a NIKON 90i microscope, and the fluorescence intensity was quantified with ImageJ software.

### Hemocyte counting

The total hemocyte counting was performed with a hemocytometer, as previously described^12,24^. Briefly, 12 h after wasp infection, larvae were bled into 20 μl PBS. The hemocyte suspension was transferred to a Neubauer-improved hemocytometer (Marienfeld) for counting under the microscope. Plasmatocytes and lamellocytes were classified based on their morphology. In total, more than 10 larvae were counted for each genotype.

### Metabolite assays

Glucose, trehalose, glycogen, and triglyceride were measured as described^81^, using the GAGO-20 kit (Sigma).

### Western Blot

Five dissected *Drosophila* muscles were pooled and lysed by lysis buffer (89802, Thermo Scientific). The following antibodies are used: Phospho-AKT (9272s, Cell Signaling), AKT (4054s, Cell Signaling), Goat anti rabbit (170–6515, Bio-Rad). Western blots were run as described^82^ and quantified by software ImageJ. Cropped gels/blots are displayed in the main figures, and full-length gels and blots are included in the Supplementary Information (see Supplementary Fig. S5).

### Starvation experiment

Second instar larvae were starved for 12 hours on apple juice agar plates (0.027 g/ml agar, 0.033 g/ml sugar, 33% apple juice concentrate, 0.002 g/ml nipagen) before allowing the wasp infect them for two hours. After 26 hours wasp infection, we tested the encapsulation rate and after 12 hours wasp infection we counted the hemocytes numbers.

### Food intake assay

Nine early third instar larvae were transferred to normal fly food containing 0.2% of Brilliant Blue FCF (80717, Sigma) and allowed to feed during one hour. Thereafter, the larvae were washed and dried and then transferred to 800 μl distilled water in an Eppendorf tube. After grinding with a pestle, the homogenates were centrifuged at maximum speed on an Eppendorf centrifuge for 15 minutes and supernatants were filtered with a 0.20 μm filter. 200 μl of the filtered solution was used to measure the absorbance at 629 nm. The larvae fed on normal food without dye were also collected as a blank control. In total, at least four independent experiments were performed.

### Quantitative PCR

Expression of *ilp2*, *ilp3* and *ilp5* was assayed by RT-qPCR, as previously described^12^. Briefly, around 15 brains from third instar larvae were dissected out and pooled for each sample. In total, at least five biological samples were assayed for each experimental group, using the following primers: *ilp2*-F1 CTCAATCCCCTGCAGTTTGT; *ilp2*-R1 CGCAGAGCCTTCATATCACA; *ilp2-F2* TGAGTATGGTGTGCGAGGAG; *ilp2-R2* GCGGTTCCGATATCGAGTTA; *ilp3-F1* ACCCCGTGAACTTCAATCAG; *ilp3-R1* GGCAGCACAATATCTCAGCA; *ilp3-F2* ACCCCGTGAACTTCAATCAG; *ilp3-R2* TGGCAGCACAATATCTCAGC; *ilp5-F1* TCAATTCAATGTTCGCCAAA; *ilp5*-R1 CGTGGAAAAGGAACACGATT; *ilp5*-F2 CGTGATCCCAGTTCTCCTGT; *ilp5*-R2 TAATCGAATAGGCCCACTGC; *Rpl32*-F1 TTCTGCATGAGCAGGACCTC; *Rpl32*-R1 GGTTACGGATCGAACAAGCG.

## Electronic supplementary material

Supplementary Information
