# Supplementary material for: Drosophila muscles regulate the immune response against wasp infection via carbohydrate metabolism
Source: Sci Rep. 2017 Nov 16;7:15713. doi: 10.1038/s41598-017-15940-2 (PMC5691183; doi:10.1038/s41598-017-15940-2)
Supplement: Supplementary file 1 — Supplementary Information [file 41598_2017_15940_MOESM1_ESM.pdf]

Supplementary Information

Drosophila muscles regulate the immune response against wasp infection via carbohydrate metabolism

Hairu Yang,[1] and Dan Hultmark[1,2]

[1]Department of Molecular Biology, Umeå University, S-901 87 Umeå, Sweden

[2]Institute of Biomedical Technology, University of Tampere, FI-33520 Tampere, Finland
Correspondence should be addressed to D.H. (dan.hultmark@ucmp.umu.se)

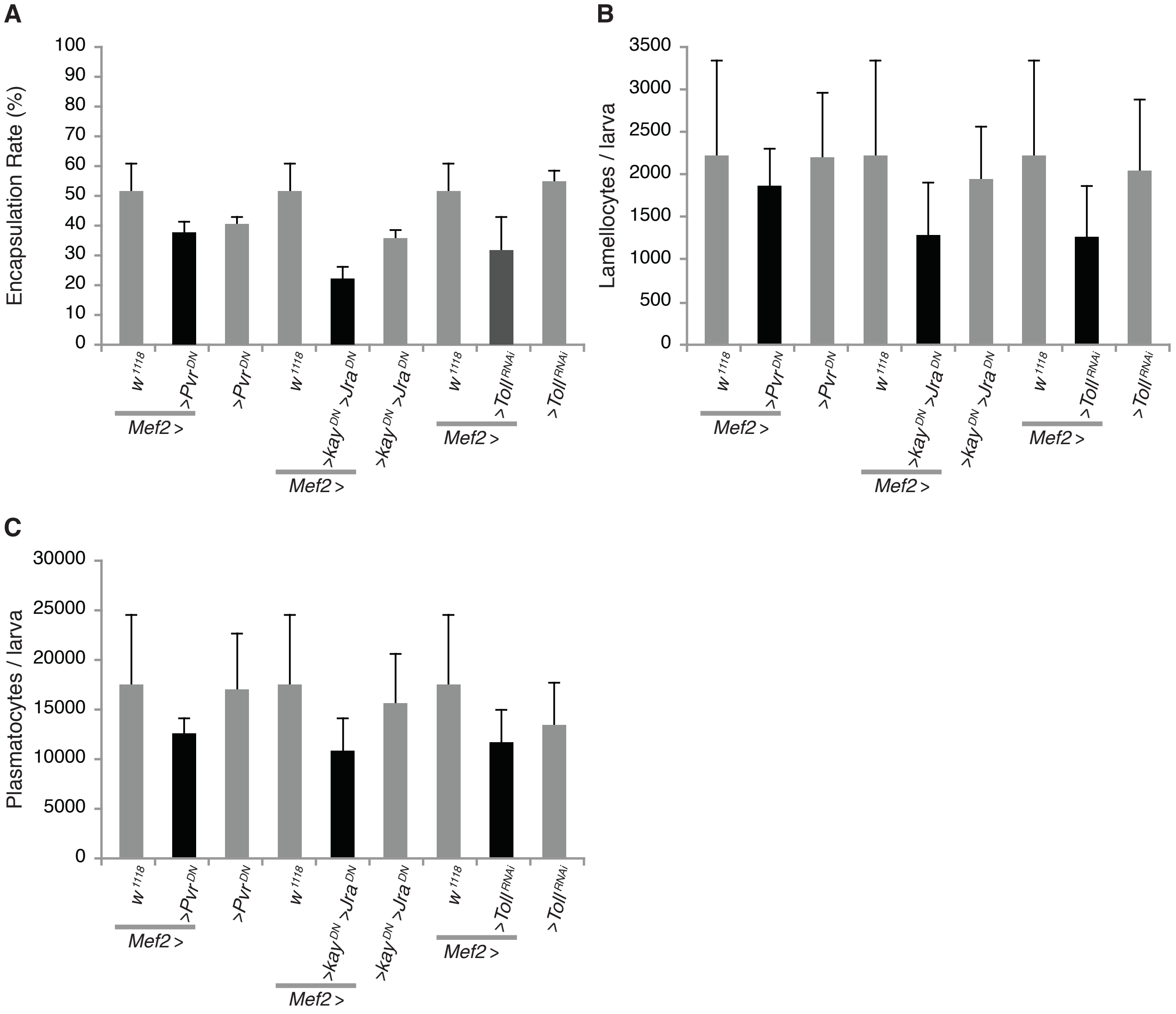

Supplementary Figure S1.

Effects on the cellular immune responses, after suppressing the PVR, JNK, and Toll signaling pathways in larval skeletal muscles.

(**A**) Encapsulation rates when suppressing the PVR, JNK, or Toll signaling pathways in muscles with the indicated genetic constructs. (**B-C**) Number of lamellocytes (**B**) and plasmatocytes (**C**) per larva after 12 h wasp infection, when suppressing the PVR, JNK, or Toll signaling pathways in muscles with the indicated genetic constructs.

Data information: Encapsulation rates were determined in at least three independent experiments, and in total at least 100 larvae were analyzed. Bars show averages and standard deviations. For hemocyte counts, at least eight larvae were analyzed for each genotype. Bars show averages and standard deviations. None of the effects seen here were statistically significant.

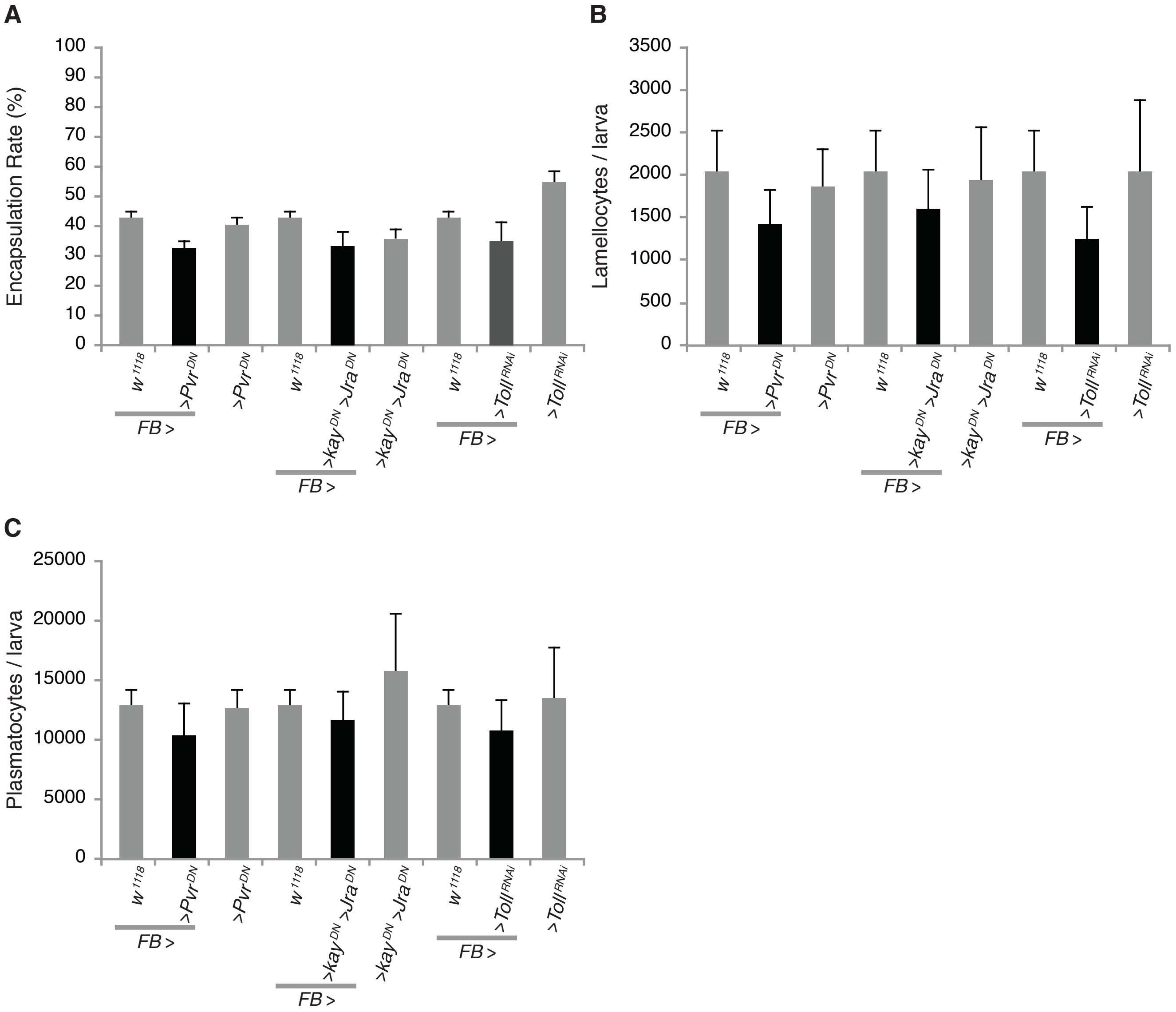

Supplementary Figure S2.

Effects on the cellular immune responses, after suppressing the PVR, JNK, and Toll signaling pathways in larval fat body.

(**A**) Encapsulation rates when suppressing the PVR, JNK, or Toll signaling pathways in fat body with the indicated genetic constructs. (**B-C**) Number of lamellocytes (**B**) and plasmatocytes (**C**) per larva after 12 h wasp infection, when suppressing the PVR, JNK, or Toll signaling pathways in fat body with the indicated genetic constructs.

Data information: Encapsulation rates were determined in at least three independent experiments, and in total at least 100 larvae were analyzed. Bars show averages and standard deviations. For hemocyte counts, at least eight larvae were analyzed for each genotype. Bars show averages and standard deviations. None of the effects seen here were statistically significant.

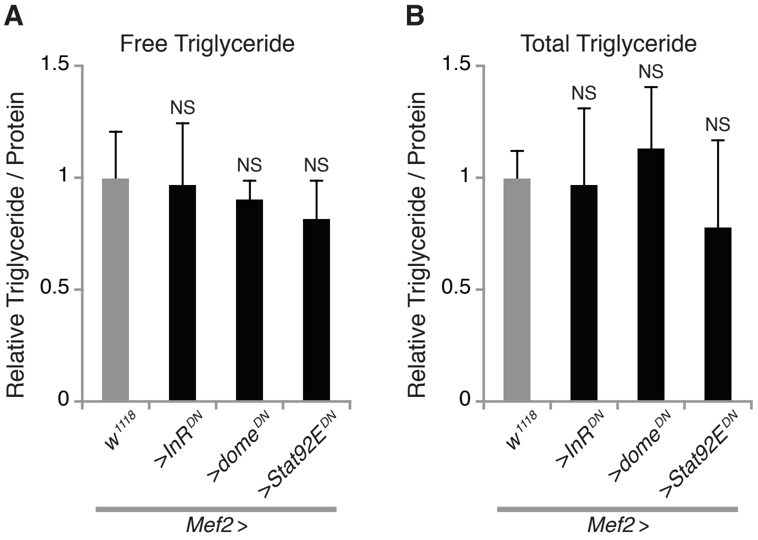

Supplementary Figure S3.

Suppression of insulin or JAK/STAT signaling in muscles does not affect free triglyceride or total triglyceride.

(**A,B**) Relative content of (**A**) free triglycerides or (**B**) total triglycerides in whole larvae, when suppressing JAK/STAT or insulin signaling respectively in muscles with the indicated genetic constructs.

Data information: For triglyceride measurements, at least three independent experiments were done. Bars show averages and standard deviations, NS: not significant.

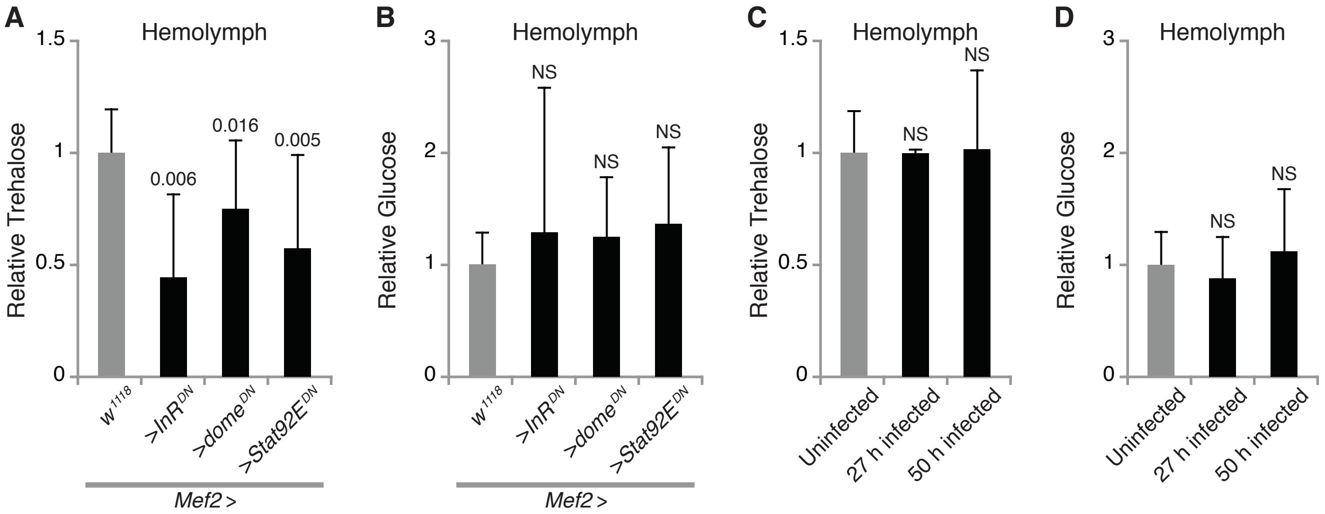

Supplementary Figure S4.

Insulin and JAK/STAT signaling in muscles, as well as wasp infection, affect trehalose in hemolymph but not free glucose.

(**A,B**) Relative content of (**A**) trehalose or (**B**) free glucose in larval hemolymph when suppressing JAK/STAT or insulin signaling respectively in muscles with the indicated genetic constructs. (**C,D**) Relative content of (**C**) trehalose or (**D**) free glucose in hemolymph of infected larvae at different times after infection.

Data information: For trehalose and glucose measurements, at least three independent experiments were done. Bars show averages and standard deviations. The *P*-values (unpaired *t*-test, unequal variance) are indicated, NS: not significant.

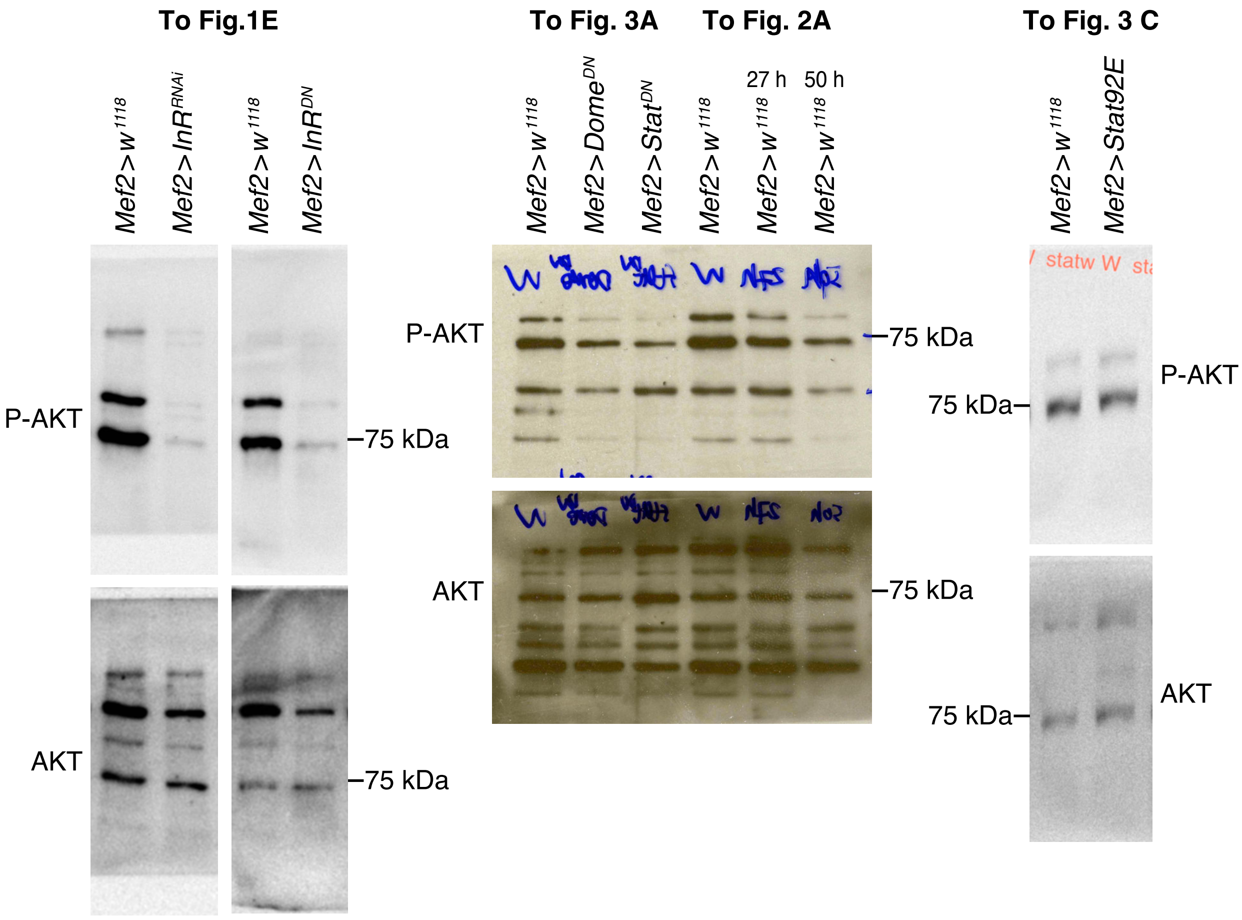

Supplementary Figure S5.

Full-length images of immunoblots, corresponding to the cropped versions shown in Fig. 1E, 2A, 3A and 3C.

These images include the entire lengths of the relevant blots. For consistency with other graphs, mirror images are shown, except for those connected to Fig. 3C. In order to preserve the linearity of the recorded signals, no processing has been done to the images, except rotation, background subtraction, and conversion to pdf file.
